# A superantigen-based MHC class II-targeted cancer immunotherapy for the treatment of acute myeloid leukemia

**DOI:** 10.1038/s41408-025-01391-w

**Published:** 2025-11-17

**Authors:** Lena Golick, Reeder M. Robinson, Leticia Reyes, Nadia St. Thomas, Kathleen Klinzing, Erin C. O’Connor, Leonardo M. R. Ferreira, Nathan G. Dolloff

**Affiliations:** 1 Department of Pharmacology and Immunology, Medical University of South Carolina, Charleston, SC USA; 2 MUSC Hollings Cancer Center, Charleston, SC USA

**Keywords:** Acute myeloid leukaemia, Cancer immunotherapy

## Abstract

The treatment of acute myeloid leukemia (AML) remains a challenge due to disease heterogeneity, which undermines efforts to develop targeted therapeutics, leaving conventional chemotherapy as the standard of care (SOC). Sialic acid binding Ig-like lectin 3 (CD33) is a myeloid cell surface glycoprotein that is highly expressed on AML blasts. However, while ~90% of AML cases express CD33, 50% of these patients harbor a single nucleotide polymorphism (SNP) that eliminates the antibody binding epitope for existing CD33-targeted antibody therapeutics. In this study, we developed an immunotherapy (M2T-CD33) that targets CD33 directly to MHC class II (MHCII) molecules on antigen presenting cells for enhanced presentation to the immune system. We found that M2T-CD33 induces a robust polyclonal anti-CD33 humoral response composed of the full immunoglobulin repertoire. M2T-CD33 induced an anti-AML response in a syngeneic mouse model that was dependent on CD4+ and CD8+ T cells. The immune response was elicited against both the full length and spliced version of CD33 and showed no evidence of toxicity at concentrations 40-fold higher than the efficacious dose. Finally, M2T-CD33 was enhanced by combinations with anti-PD-1 therapy. These experiments demonstrate the preclinical potential of M2T-CD33 in AML and emphasize the importance of MHCII for cancer immunotherapy.

## Introduction

Acute myeloid leukemia (AML) is a hematological malignancy characterized by proliferation and clonal expansion of immature myeloid cells in the bone marrow and blood. AML disrupts normal hematopoiesis in the bone marrow leading to pancytopenia, susceptibility to infections and bleeding, and other complications. In the United States there are ~20,800 new cases and AML is responsible for 11,220 deaths per year [1]. According to the National Cancer Institute’s Surveillance, Epidemiology, and End Results database, the 5-year survival rate for AML has improved since 1980, but remains at only ~28%, and for patients >70 years old, survival drops to a mere 5% [2]. AML consists of multiple disease subtypes and disease heterogeneity characterized by a variety of cytogenetic abnormalities and genetic mutations [3, 4]. These features make it challenging to develop a generalized targeted therapeutic, leaving conventional chemotherapy as the current standard of care (SOC) [5–7]. The predominant focus for AML treatments has been for adult (aAML), since the incidence of AML peaks in the elderly. However, AML is also the fifth most common malignancy in children [8]. Pediatric AML (pAML) currently has a 5-year survival rate of 67-68% [9], and a survival rate after relapse of just 30-40% [10]. Additionally, while aAML is thought to be caused by an accumulation of somatic mutations, these mutations are relatively rare in pAML. It is much more common for pediatric patients to exhibit cytogenetic abnormalities that lead to de novo AML [11]. These differences highlight the need for new treatment modalities that target both adult and pediatric populations through a unifying pathway. An immunotherapy platform that targets a common cell surface antigen in a genetics-independent manner would be an ideal solution.

Major Histocompatibility Complexes (MHC) play crucial roles in the immune response by presenting antigens to T cells. MHC is divided into two classes, MHC class I (MHCI) and MHC class II (MHCII), each of which presents antigens to different subsets of T cells. MHCI directly interacts with CD8+ T cells, whereas MHCII activates CD4+ helper T cells. Much of the cancer immunotherapy field has focused on the MHCI-CD8 axis, given the cytotoxic phenotype of CD8+ T cells and their ability to recognize and eliminate tumor cells directly. However, more recent evidence shows that CD4+ T cells are essential for orchestrating an effective cancer immunotherapy response [12–15]. CD4+ T cells support activation and proliferation of CD8+ T cells along with other immune cells, such as macrophages, B cells, and NK cells, and skew the immune system toward a more tumor-suppressive phenotype [16–18]. This suggests that targeting the MHCII-CD4+ axis could have powerful immune modulating effects. The clinical validation of LAG-3 inhibitors and soluble LAG-3 (IMP321/eftilagimod alpha [19]) supports this therapeutic concept, given that MHCII is the binding target of LAG-3 [20–22]. Furthermore, MHCII expression is limited to professional APCs whereas MHCI is ubiquitously expressed on APCs but also virtually all cells throughout the body. Innovative strategies that directly engage the MHCII-CD4+ pathway are therefore of significant interest in the development of next-generation cancer immunotherapies and an area of intense investigation by our team and others [23–25].

Sialic acid binding Ig-like lectin 3 (CD33) is a cell surface glycoprotein that is expressed by AML and normal myeloid lineage cells [26]. It is a member of the Siglec family, characterized by cell-cell interactions through binding of sialylated glycans. CD33 consists of two extracellular domains, an Ig-like V (IgV) domain and an Ig-like C (IgC) domain, a transmembrane domain, an immunoreceptor tyrosine-based inhibitory motif (ITIM) domain, and an ITIM-like domain. Although the role of CD33 is not well understood, it is thought to inhibit cellular activation and proliferation upon ligand binding [27]. CD33 is normally expressed on myeloid cells and is present in ~85-90% of both pAML and aAML cases [28]. CD33 has been the target of multiple immunotherapy strategies that target AML in preclinical and clinical research [26], but only one CD33-targeted therapeutic has been FDA approved for pAML—gemtuzumab ozogamicin/MYLOTARG (GO). However, there are multiple disadvantages to GO. GO is an antibody-drug conjugate (ADC) comprised of an anti-CD33 monoclonal antibody linked to a cytotoxic calicheamicin payload. The binding epitope for GO is on the IgV domain, whereas 50% of CD33-positive AML patients express a single nucleotide polymorphism (SNP), rs12459419 C > T, that leads to a CD33 splice variant (SV) lacking the IgV domain [29]. This renders GO ineffective in half of the patients eligible for its use because the CD33 SV lacks the gemtuzumab binding epitope. Furthermore, premature cleavage of the calicheamicin linker can cause off-target effects. Usage of calicheamicin also increases the risk of veno-occlusive disease (VOD) in patients that receive hematopoietic stem cell transplants (HSCTs) [30]. Another potential issue with GO is resistance to calicheamicin. Previous studies have shown that the expression of P-glycoprotein (Pgp) on AML blasts can lead to drug efflux and reduce the efficacy of GO [31]. Lastly, GO has only shown a modest improvement in a clinical trial comparing pAML patients receiving standard chemotherapy vs. chemotherapy with the addition of GO. Event free survival (EFS) was significantly improved for patients receiving GO vs. standard chemotherapy, but there was no difference in overall survival (OS). When patients were stratified into low-risk, intermediate-risk, and high-risk groups, none showed a significant increase in event free survival [32]. These drawbacks highlight the critical need for a safer and more effective therapeutic that targets CD33-positive AML.

Our lab constructed an MHC class II-targeted (M2T) immunotherapy conjugate that delivers the extracellular domain (ECD) of CD33 to APCs for presentation to the immune system. The MHCII targeting component incorporates structural design elements taken from naturally occurring proteins called bacterial superantigens (SAgs). Produced by certain strains of bacteria (e.g., *Staphylococcus aureus* and *Streptococcus pyogenes*), SAgs function by binding to and crosslinking MHCII molecules and T cell receptor β chains (TCRVβ). In addition to crosslinking MHCII and TCRVβ, they provide costimulatory signaling through the CD28/B7 pathway to induce full T cell activation [33, 34]. This leads to mature T cell proliferation, overproduction of cytokines, and eventually T cell anergy [35, 36]. SAgs include staphylococcal enterotoxin B (SEB), staphylococcal enterotoxin A (SEA), toxic shock syndrome toxin-1 (TSST-1), and streptococcal mitogenic exotoxin Z (SMEZ). Building on the work of others [37, 38], our approach utilizes SMEZ-2, the most potent SAg [37]. Crystallography and protein modeling have revealed key functional regions that mediate the binding of SAgs to MHCII and the TCR complex. From these studies, we know that SAg binding to the MHCII β chain occurs via a zinc-dependent high affinity site forming a complex that is stable for >40 h [35, 39]. We reasoned that the MHCII binding motif of SAgs could be used to deliver TAAs directly to MHCII on APCs for subsequent presentation to CD4+ T cell populations. Previous work by others has in fact demonstrated the potential of this approach to stimulate immunity against an ovalbumin antigen using a modified SAg [40]. Amino acids W75, K182, and D42 in SMEZ-2 form contacts with TCRVβ, and modification of these residues eliminated TCR interaction and the induction of a cytokine storm while preserving the ability to bind to MHCII. Their work showed an increase in the humoral response to antigens conjugated to modified SMEZ-2 [38]. Antibody-based MHCII-targeted approaches have shown similar ability against influenza [41] and the SARS-CoV-2 Spike protein for the treatment of COVID [42]. M2T-CD33 uses a similar design, conjugating the CD33 ECD to modified SMEZ-2.

In this study we evaluated the preclinical potential of M2T-CD33. We tested the MHCII specificity of M2T-CD33 in vitro and established a syngeneic immunocompetent mouse model for in vivo studies. M2T-CD33 treatment induced significantly higher anti-CD33 antibody titers compared to CD33 alone and led to increased overall survival. Although M2T-CD33 was effective against CD33-positive AML cells, treatment did not affect normal myeloid cells in mice. Antibodies induced by M2T-CD33 treatment were also able to recognize both full length CD33 and CD33 SV, compared to existing anti-CD33 monoclonal antibodies (mAbs) which could only recognize full-length CD33. Furthermore, anti-leukemic efficacy was dependent on CD4+ and CD8+ T cells and was enhanced by combination with an anti-PD-1 therapy. These experiments demonstrate the preclinical potential of M2T-CD33 as an immunotherapy for CD33-positive AML.

## Materials and Methods

### Cell lines and reagents

Cell lines (HL-60, MOLM-14, THP-1, Kasumi-1, MV4-11, Daudi, Jurkat, C1498, HEK293T) were purchased from American Type Culture Collection (ATCC, Manassas, VA, USA). Human peripheral blood mononuclear cells (PBMCs) were purchased from STEMCELL Technologies (Cambridge, MA, USA). Mouse splenocytes and bone marrow were harvested from C57BL/6 J mice. Cells were cultured with either RPMI-1640 (Cytiva, Cat #SH30027.01) or DMEM (ATCC, Cat #30-2002) according to manufacturer recommendations and supplemented with 10% heat-inactivated fetal bovine serum (Gibco, Cat #A5670701) and 1% penicillin (10,000 IU/mL)/streptomycin (10,000 µg/mL) (Corning, Cat #30-002-CI) and kept at 37°C and 5% CO2. IFN-γ stimulation was done with 20 ng/mL of IFN-γ for 48 h (Gibco, Cat #PMC4034). Expi293F Expression System was purchased from Thermo Fisher Scientific (Cat #A14635). Expi293F cells were cultured following manufacturer’s instructions in Expression Medium and kept at 37°C and 8% CO2 shaking at 124 rpm.

### Flow cytometry

Cells were collected, counted, and 0.5-1 × 10^6^ cells per sample were used for flow. 10 × 10^6^ cells per sample were used for bone marrow analysis. Blood samples were treated two times with ACK lysing buffer (Gibco, Cat #A1049201) following manufacturer’s protocol. Samples were then washed with 1 mL ice-cold FACS buffer (1X PBS + 1% FBS + 150 µM CaCl2, pH 7.4 or 1X PBS + 1% FBS, pH 7.4). Centrifugation steps were carried out at 2500 rpm for 3 min at 4°C. Cells were incubated on ice in primary antibody or protein diluted in a total of 100 µL FACS buffer for 20 min covered from light. Cells were then washed with 500 µL FACS buffer, spun, resuspended in 300-500 µL FACS buffer, and moved to flow tubes for analysis. If a secondary antibody was used, the incubation step was repeated with secondary antibody before moving to the final resuspension step. Human or mouse seroblock (Bio-Rad, Cat #BUF070B or #BUF041B) was used before primary antibody, or after protein and before secondary antibody, for human PBMCs, mouse blood, and mouse splenocytes. 5 µL of seroblock was added per sample in 50 µL total and incubated covered on ice for 5 min. Antibody diluted in 50 µL was added on top of seroblock without washing. Bone marrow cells were blocked with Universal Fc Receptor Blocker (Cat #NB335) from Innovex Biosciences (Richmond, CA, USA), for 30 min covered on ice prior to staining. Recombinant proteins were detected with His-Tag Rabbit mAb AF488, mouse plasma was detected with Anti-Mouse IgG PE, and recombinant antibodies were detected with Protein L PE. Flow cytometry was run on a NovoCyte (ACEA Biosciences; San Diego, CA, USA), an LSRFortessa X-20 (BD Biosciences; Franklin Lakes, NJ, USA), or a CytoFLEX LX (Beckman Coulter; Indianapolis, Indiana, USA) and analyzed using FlowJo software (version 10.10.0). PE Anti-Human CD33 (Cat #366608), PE Mouse IgG1k Isotype Control (Cat #400114), PE Anti-Mouse CD19 (Cat #152408), FITC Anti-Mouse CD45 (Cat #103108), PE-Cy5 Anti-Mouse CD4 (Cat #100514), PE Anti-Mouse PD-L1 (Cat #124307), BV421 Anti-Mouse CD34 (Cat #152207), APC Anti-Mouse CD16/32 (Cat #101325), PE Anti-Mouse Lineage Cocktail (Cat #133303), AF700 Anti-Mouse Sca-1 (Cat #108141), PE-Cy7 Anti-Mouse c-Kit (Cat #105813), BV510 Anti-Mouse CD45 (Cat #103137), PerCP-Cy5.5 Anti-Mouse CD3 (Cat #100327), PE-Cy7 Anti-Mouse/Human CD44 (Cat #103029), AF700 Anti-Mouse CD62L (Cat #104426), APC Anti-Mouse PD-1 (Cat #135209), BV421 Anti-Mouse TIM-3 (Cat #119723), and BV605 Anti-Mouse LAG-3 (Cat #125257) were purchased from BioLegend (San Diego, CA, USA). BV421 Anti-Rabbit IgG (Cat #565014), FITC Anti-Human HLA-DR (Cat #347363), and FITC Mouse IgG2ak Isotype Control (Cat #554647) were purchased from BD Biosciences (Franklin Lakes, NJ, USA). Anti-Human TCR alpha/beta PE (Cat #12-9986-42), Anti-Human CD19 PE (Cat #12-0199-42), Anti-Mouse IgG PE (Cat #12-4010-82), Anti-Mouse CD8a PE (Cat #12-0081-82), Anti-Rabbit IgG AF488 (Cat #R37116), LIVE/DEAD Fixable Near IR (876) Viability Kit (Cat #L34980), and Anti-Mouse CD4 FITC (Cat #11-0042-82) were purchased from Invitrogen (Thermo Fisher Scientific; Waltham, MA, USA). His-Tag Rabbit mAb AF488 (Cat #14930S), Anti-Human CD3 PE (Cat #46233S), and Rat IgG2b Isotype Control PE (Cat #27426S) were purchased from Cell Signaling Technology (Danvers, MA, USA). Anti-Mouse CD3 PE (Cat #FAB4841P) was purchased from R&D Systems (Minneapolis, MN, USA). Anti-Mouse CD33 (Cat #ab270990) was purchased from Abcam (Waltham, MA, USA). Recombinant Protein L PE (Cat #11044-H07E-P) was purchased from Sino Biological (Wayne, PA, USA). Mouse plasma was analyzed using the Th1/Th2 Th17 Cytometric Bead Array Kit (Cat #560485) from BD Biosciences following the manufacturer’s instructions.

### Cloning and recombinant protein production

Amino acid sequences for hCD33, mCD33, SMEZ-2, gemtuzumab, lintuzumab, and various MHCII proteins were codon optimized using GenScript (Piscataway, NJ, USA). MHCII proteins were expressed as IgG1 Fc Knob-in-Hole fusions. The codon optimized DNA sequences were purchased from GenScript in pUC57 and subcloned into pcDNA3.4 using NheI and XhoI restriction sites with an N-terminal secretion signal and C-terminal 6X-His tag, except monoclonal antibodies (mAbs), which used NheI and AgeI with an N-terminal secretion signal. Amino acid changes were introduced by site-directed mutagenesis. Proper ligation of each gene was confirmed by DNA sequencing through Eurofins Genomics. Proteins were expressed in Expi293F cells following manufacturer’s instructions. For mAbs, heavy and light chain DNA sequences were mixed at a 1:2 ratio. Cell supernatant was collected between 50 and 60% viability or on Day 7 after transduction. Supernatant was purified using an AKTAprime plus and a HisTrap excel column (Cat #17371206), or a HiTrap rProtein A FF column for mAbs (Cat #17508001) (Cytiva; Marlborough, MA, USA). The HisTrap excel column was equilibrated with buffer A (20 mM sodium phosphate, 0.5 M NaCl, pH 7.4), washed with buffer A containing 25 mM imidazole, and eluted with buffer A containing 500 mM imidazole. The HiTrap Protein A HP column was equilibrated with buffer B (20 mM sodium phosphate, pH 7.0), washed with buffer B, and eluted with buffer C (100 mM sodium citrate, pH 3.0) into tubes containing neutralization buffer (1 M Tris-HCl, pH 9.0). Fractions were run on an NuPAGE 4-12% Bis-Tris Gel (Invitrogen, Cat #NP0336BOX) at 200 V for 48 min, Coomassie Blue stained, and visualized for protein purity. Pooled fractions were dialyzed in 1X PBS overnight at 4°C. Dialyzed protein was concentrated using Amicon Ultra centrifugal filters (Cat #UFC901024) from Millipore Sigma (Burlington, MA, USA) and concentrations were calculated by nanodrop using A_280_ and the molecular weight and extinction coefficient estimated using Expasy ProtParam. Concentrated proteins were frozen in pellets in liquid nitrogen and stored at -80°C.

### Western blotting

Proteins were diluted to 200 ng with Milli-Q H_2_O and 10X SDS sample buffer containing β-mercaptoethanol (Fisher Scientific, Cat #BP176-100) was added. Samples were boiled at for 5 min, then loaded on NuPAGE 4−12% Bis-Tris Gel (Invitrogen, Cat #NP0336BOX) along with protein standards (Bio-Rad, Cat #161-0374). Samples were run at 200 V for 48 min in 1X NuPAGE MOPS SDS Running Buffer (Invitrogen, Cat #NP0001). Gels were transferred to polyvinylidene difluoride (PVDF) membranes at 0.3 A for 2 h in 1X transfer buffer containing 25 mM trizma base, 192 mM glycine, and 20% methanol. Membranes were blocked for 30 min in 5% milk TBST on a rocker at room temperature. Membranes were incubated in Anti-6X His primary antibody (Invitrogen, Cat #MA1-21315) in 5% milk TBST rocking overnight at 4°C. The membranes were washed 3X with TBST and incubated in Anti-Mouse IgG HRP secondary antibody (Invitrogen, Cat #31430) in 5% milk TBST for 2 h rocking at room temperature. Membranes were washed again 3X with TBST and then developed using ECL (Thermo Fisher Scientific, Cat #32209).

### Enzyme-Linked Immunosorbent Assay (ELISA)

Antibody titers were calculated by indirect ELISA. 96-well MaxiSorp Plates (Thermo Fisher Scientific, Cat #430341) were coated with 10 ug/mL of recombinant hCD33 ECD or mCD33 ECD diluted in coating buffer (50 mM sodium bicarbonate/carbonate, pH 9.6), covered with plastic wrap, and incubated at 4°C overnight. Coating buffer was removed from plates by flicking out contents and blotting on paper towel. Wells were washed three times with 100 µL PBST. 200 µL per well of blocking buffer (50 mM Tris-HCl, 140 mM NaCl, 1% BSA, pH 8.0) was added incubated at room temperature for 30 min. The wash step was repeated, 100 µL of sample was added per well diluted in assay diluent (50 mM Tris-HCl, 140 mM NaCl, 1% BSA, 0.05% Tween-20, pH 8.0), and plates were incubated at 37°C for 1 h. After incubation, the wash step was repeated and Anti-Mouse IgG HRP secondary antibody (Invitrogen, Cat #31430) was diluted 1:100,000 in assay diluent and incubated with 100 µL per well for 1 h at room temperature. Wells were washed five times with 100 µL PBST and 100 µL per well of TMB substrate (Thermo Fisher Scientific, Cat #34021) was added for 15–30 min at room temperature, protected from light. 100 µL of stop solution (0.18 M sulfuric acid) was added to each well and plates were read at 450 nM on a SpectraMax i3 plate reader (Molecular Devices; San Jose, CA, USA). Antibody titers were interpolated from a standard curve in GraphPad Prism software (version 10.4.1). Total immunoglobulin subtypes were determined using a mouse Ig isotyping uncoated ELISA kit (Invitrogen, Cat #88-50630-88). Anti-CD33-specific immunoglobulins were determined modifying the ELISA kit protocol by coating with 10 µg/mL CD33 instead of capture antibodies, adding capture antibodies as secondary antibodies after mouse plasma, and then detecting with Anti-Rat IgG-HRP (Invitrogen, Cat #62-9520).

### Lentiviral transfection and stable expression of C1498-hCD33

The codon optimized hCD33 sequence was subcloned into pLJM1 using AfeI and EcoRI as restriction sites. Proper ligation of each gene was confirmed by DNA sequencing through Eurofins Genomics. HEK293T cells were transfected with 15 µg midi-prepped DNA, 7.5 µg pCMV-dR8.91, and 1.5 µg pCMV-VSV-G using lipofectamine 2000 (Invitrogen, Cat #11668027). Virus was collected 3 days later, filtered, and stored at -80°C. C1498 cells were transduced with 1 mL of hCD33 virus in medium supplemented with 8 µg/mL of polybrene (Millipore Sigma, Cat #TR-1003-G) and centrifuged at 2250 rpm for 2 h at room temperature before being placed in a cell incubator. Antibiotic selection was started 3 days later with 10 µg/mL of puromycin (Gibco, Cat #A1113803) and cells were subcultured until stable transduction was established.

### C1498 syngeneic AML mouse model

All animal experiments were approved and conducted in accordance with the Medical University of South Carolina (MUSC) Institutional Animal Care and Use Committee (IACUC, Protocol #IACUC-2020-00969-1). Animals were randomly assigned to treatment groups. Six to eight week old female C57BL/6 J mice (Jackson Laboratory; Bar Harbor, ME, USA) were injected with 0.05 or 0.5 × 10^6^ C1498-hCD33 cells by lateral tail vein in 100 µL of 1X PBS. M2T-CD33 and controls were injected at the concentration indicated by lateral tail vein in 100 µL of 1X PBS for the schedule described in each experiment. Mice were bled by tail vein and 100 µL of blood was added to 20 µL of EDTA (50 mM) and then 100 µL of 20mmM EDTA was added. Blood was centrifuged at 3,600 x g for 15 min at 4°C and plasma was stored at -80°C. For the depletion experiment, Rat IgG2b Isotype antibody (Cat #BE0090), anti-CD4 antibody (Cat #BE003-1), or anti-CD8 antibody (Cat #BE0061) were injected intraperitoneally at an initial dose of 200 µg 2 days before injection of C1498-hCD33 cells and then 100 µg every 4 days for the duration of the experiment. For combination with cytarabine, 2 mg of cytarabine (Millipore Sigma, Cat #1162002) per mouse dissolved in 100 µL 1X PBS was injected intraperitoneally on Days 1, 2, 3, and 4. Mice were weighed two times a week to track changes in body weight. Complete blood count with differential and plasma chemistry analysis was completed by MUSC Division of Lab Animal Resources (DLAR). For combination with an immune checkpoint inhibitor (ICI), 50 µg of Rat IgG2a Isotype antibody (Cat #BE0089) and 50 µg Syrian Hamster IgG Isotype antibody (Cat #BE0087) or 100 µg of anti-PD-1 antibody (Cat #BE0146) was injected intraperitoneally two times a week starting 1 day after injection of C1498-hCD33 cells. ICI was injected for 3 weeks and then given a 2 week break, followed by a weekly maintenance dose for 7 weeks. For the cytometric bead array, 100 μg of anti-CD40 antibody (Cat #BE0016-2) was injected intraperitoneally two times, 2 weeks apart. All antibodies were purchased from Bio X Cell (Lebanon, NH, USA). Animals were monitored daily by a blinded veterinary technician.

### Complement Dependent Cytotoxicity (CDC) assay

C1498-hCD33 and C1498 parental cells were incubated with varying dilutions of M2T-CD33 treated or Naïve C57BL/6 J plasma and baby rabbit complement (MP Biomedicals, Cat #642961). After 48 h, cell viability was determined using CellTiter-Glo (Promega, Cat #G9683) and plates were read at 470 nm on a SpectraMax L plate reader (Molecular Devices; San Jose, CA, USA).

### Aggregation assay

Proteins were diluted to 100 µg/mL and analyzed using the PROTEOSTAT Protein aggregation assay following the manufacturer’s instructions (Enzo, Cat #ENZ-51023-KP050). The plate was read using an excitation setting of 550 nm and an emission filter of 600 nm.

### Enzyme-Linked Immunospot (ELISpot)

IFN-γ ELISpot was performed using the BD ELISpot Mouse IFN-γ ELISpot Set following the manufacturer’s instructions (Cat #551083). Briefly, plates were coated with capture antibody diluted in coating buffer (1X PBS) and incubated at 4°C overnight. Wells were aspirated and washed once with blocking buffer (RPMI-1640, 1 mM HEPES, 1% NEAA (Corning, Cat #25-025-Cl), 1% sodium pyruvate (Corning, Cat #25-000-CI), 10% FBS, 1% penicillin/streptomycin) and then incubated with blocking buffer for 2 h at room temperature. Recombinant hCD33 ECD or bovine serum albumin (BSA, Millipore Sigma, Cat #A2153) as a negative control were diluted in Mouse T Cell Medium (RPMI-1640, 1 mM HEPES, 1% NEAA, 1% sodium pyruvate, 10% FBS, 1% penicillin/streptomycin, 55 µM β-mercaptoethanol) and added to the wells at a final concentration of 50 µg/mL with 50 ng/mL of IL-7 (STEMCELL Technologies, Cat #78196) and IL-15 (BioAbChem, Cat #44-IL15). Splenocytes were diluted in Mouse T Cell Medium and 0.5 × 10^6^ cells were added per well for a total volume of 100 µL per well. Plates were placed in the incubator for 48 h. Wells were aspirated and washed twice with 200 µL per well Milli-Q H_2_O, flicking out the contents and blotting on a paper towel after each wash. Wells were then washed three times with PBST. Detection antibody was diluted in 1X PBS + 10% FBS, added to wells, and incubated for 2 h at room temperature. Wells were washed three times with PBST. Enzyme conjugate was diluted in 1X PBS + 10% FBS, added to wells, and incubated for 1 h at room temperature. Wells were washed four times with PBST and then two times with 1X PBS. BD ELISpot AEC Substrate Set (Cat #551951) was added to wells for 1 h at room temperature. Plates were air-dried overnight without lid and plastic tray. Plates were then sent to Cellular Technology Limited (Shaker Heights, Ohio, USA) for analysis of spot-forming cells (SFCs). Plates were quantified by averaging duplicate wells, subtracting the mean number of spots from the matching BSA wells, and reported per 1 × 10^6^ splenocytes.

### Statistical analysis

All statistical analyses were performed using GraphPad Prism software (version 10.4.1). Statistical significance was determined as indicated in the figure legends. Power analyses were conducted by staff biostatisticians at the MUSC Biostatistics Shared Resource to determine experiment sample size.

## Results

### In vitro characterization of M2T-CD33, an MHCII-targeted immunotherapy

**Fig. 1:**
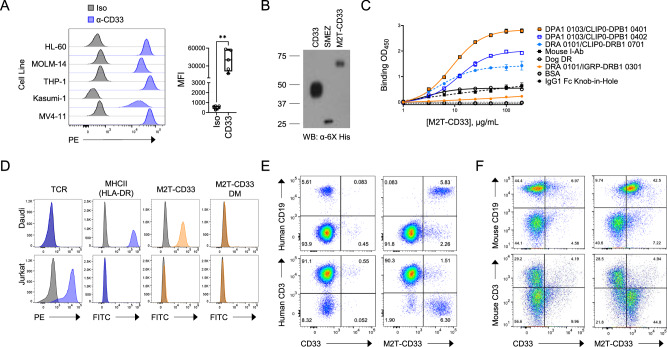
M2T-CD33 binding is specific for professional antigen presenting cells expressing MHCII. **A** Flow cytometry of various AML cell lines showing CD33 cell surface expression with median fluorescence intensity (MFI) quantification. Significance was determined by Mann-Whitney test, **P < 0.01. **B** Western blot for anti-6X His tagged recombinant proteins. **C** ELISA for M2T-CD33 binding to various MHCII alleles, including human, mouse, and dog. IgG1 Fc Knob-in-Hole as a control for MHCII alleles constructed with IgG1 Fc stalk. BSA as negative control. **D** Flow cytometry of Daudi and Jurkat cells showing TCR and MHCII (HLA-DR) expression, M2T-CD33 binding, and M2T-CD33 double mutant (DM) binding. **E** Flow cytometry of human PBMCs (healthy donor #1) gating on CD3 or CD19 and then comparing binding of CD33 vs. M2T-CD33. **F** Flow cytometry of mouse splenocytes gating on CD3 or CD19 and then comparing binding of CD33 vs. M2T-CD33.

We set out to develop an AML targeted immunotherapy approach that stimulates presentation of AML antigens and instructs the immune system to target leukemic blasts. We first verified that CD33 is highly expressed on various human AML cell lines to confirm it as a therapeutic target (Fig. 1A**)**. We then created a CD33 immuno-conjugate by cloning the ECD of CD33 (AAs 18-259) to the N-terminus of modified SMEZ-2 M1 using a single GGGGS linker (Fig. 1B). We refer to this molecule as M2T-CD33 hereafter. We optimized small-scale production and purification methods in mammalian cell culture and used in silico drug developability methods to identify potential liabilities in the protein sequence. This identified two residues (C42 in the SMEZ-2 molecule and D246 in CD33) as potential liabilities that upon substitution reduced protein aggregation, thereby improving stability, purity, and batch reproducibility of the therapeutic product (Fig. S1A). Crystallographic studies have shown that SAgs bind outside the highly polymorphic peptide binding pocket of MHCII [43]. This suggests that their binding is unaffected by the many human leukocyte antigen (HLA) polymorphisms found in the population that map to the binding groove [44]. To directly test this hypothesis, we expressed a variety of MHCII protein complexes from human, mouse, and dog and conducted binding assays to evaluate binding of M2T-CD33 in vitro. For mouse, we used the I-Ab allele that is expressed in C57BL/6 J mice to assess whether this model could be used for our in vivo studies. For human proteins, we also used multiple MHCII isoforms (*HLA-DP* and *HLA-DR*) with different polymorphisms found across human populations. For example, the DPA1*0103/DPB1*0401 is one of the most common MHCII molecules found worldwide with expression in ~45% of individuals of European descent [45]. We found that M2T-CD33 bound with high affinity and specificity to all MHCII proteins compared to the control BSA and IgG1 Fc knob-in-hole stalk that was used to stabilize recombinant MHCII complexes (Fig. 1C). Of note the strongest binding was observed with the aforementioned DPA1*0103/DPB1*0401. These data demonstrate the high affinity binding of the recombinant M2T-CD33 conjugate to multiple MHCII isoforms and polymorphisms from humans and other species in vitro. This confirms the platform’s ability to bind a diversity of MHCII molecules across patient populations with highly polymorphic HLA genes.

We next demonstrated the specificity of M2T-CD33 binding to native MHCII expressed on live APCs. B cells are major MHCII-positive professional APCs, whereas T cells are typically MHCII-negative except for when activated or in AIDS pathogenesis [46]. We performed flow cytometry using a B cell lymphoma cell line (Daudi), which is MHCII (HLA-DR)-positive. For a negative control, we used a T cell lymphoma cell line (Jurkat), which is MHCII negative and TCR-positive (Fig. 1D). Consistent with our hypothesis, we found that M2T-CD33 bound only to B cells but not T cells, confirming specific binding of the chimeric protein to MHCII-positive cells. This result also confirmed successful elimination of TCR binding, which was accomplished by mutation of three residues in the TCR binding interface of SMEZ-2 and critical for disarming the overactivation and immune toxicity of the wild-type SAg. To further confirm that M2T-CD33 binding to B cells was through MHCII, we produced an M2T-CD33 double mutant (DM) through site-directed mutagenesis and introduced two mutations in the MHCII binding domain of SMEZ-2 (H202A D204A). M2T-CD33 DM did not bind to T cells or B cells, thereby confirming the specificity of the interaction (Fig. 1D). We also confirmed binding of M2T-CD33 to primary CD19-positive B cells but not primary CD3-positive T cells from PBMCs collected from healthy human donors (Fig. 1E, Fig. S1B). Additionally, we showed binding of M2T-CD33 to mouse CD19-positive B cells but not CD3-positive T cells harvested from C57BL/6 J mouse splenocytes (Fig. 1F), further demonstrating the highly conserved cross-species binding of SMEZ-2.

### M2T-CD33 induces a CD33-specific immune response and anti-AML activity in vivo

**Fig. 2:**
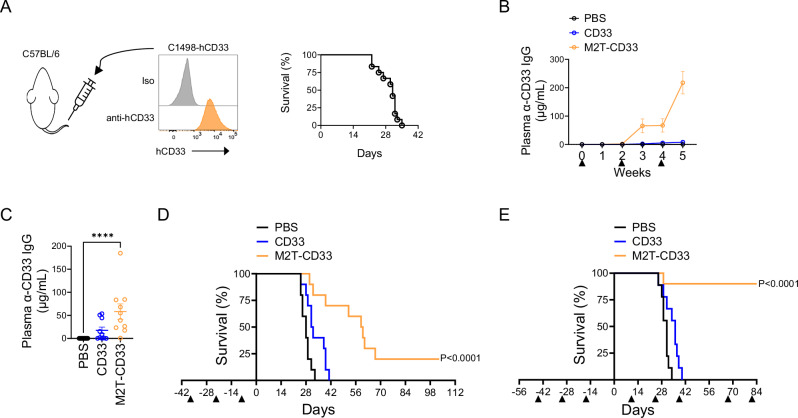
M2T-CD33 induces a polyclonal humoral response and has anti-tumor efficacy in an in vivo prophylactic model. **A** Flow cytometry of CD33 expression on C1498-hCD33 cells prior to injection. Representative survival of the model on right. **B** ELISA of anti-CD33 IgG over time after injection with M2T-CD33 or controls. Proteins were injected at a dose of 5 µg per mouse as indicated by triangles. **C**, **D** Mice were injected with 5 µg M2T-CD33 or controls as indicated by triangles and then challenged 1 week later with 0.5 × 10^6^ C1498-hCD33 cells (Day 0; *N* = 10). **C** ELISA of anti-CD33 IgG 1 week after third M2T-CD33 dose. Error bars represent standard error of the mean (SEM). Significance determined by Kruskal-Wallis test and Dunn’s multiple comparisons test, ****P < 0.0001. **D** Kaplan-Meier survival curves. Significance was determined by log-rank Mantel-Cox test, compared to vehicle control. **E** Mice were injected with 5 µg M2T-CD33 or controls as indicated by triangles and then challenged 2 weeks later with 0.5 × 10^6^ C1498-hCD33 cells (Day 0). The therapeutic regimen was then repeated every 4 weeks (*N* = 9–10). Kaplan–Meier survival curves shown. Survival significance was determined by log-rank Mantel-Cox test, compared to control.

To evaluate the efficacy of the MHCII-targeted approach in vivo, we utilized the C1498 syngeneic immunocompetent mouse model of AML [47]. C1498 cells were injected systemically via the lateral tail vein, which induces the development of AML with pathologic features that closely resemble the human pathology, including bone marrow colonization by leukemic blasts, formation of solid tumors, and ultimately death (Fig. 2A). We expressed human CD33 (hCD33) in C1498 cells for two reasons (Fig. 2A). Firstly, there are significant differences in mouse and human CD33 including myeloid lineage expression patterns and sialic acid binding [48, 49]. In fact, we found that mouse and human AML blasts differed with respect to CD33 expression. C1498 and MLL-AF9 mouse AML cells were negative or showed weak expression (Fig. S1C), whereas human AML shows high density of cell surface CD33 in a significant percentage of patients. Secondly, mouse and human CD33 share only 54% sequence identity, and so we reasoned it was best to construct a therapeutic targeting the human sequence for preclinical efficacy studies as this would eventually be the clinical candidate that would progress to studies in humans. Dose level and route of administration were selected based on early dosing studies. Mice were dosed every other week with M2T-CD33 at a dose of 5 µg (0.1 nmol). We noted a delayed onset of antigen-specific immunity. M2T-CD33 induced significant levels of anti-CD33 IgG (Mean ± SEM = 65.48 ± 24.7 µg/mL) in the blood of mice 1 week after the second dose and 21 days after the first dose compared to mice dosed with the PBS vehicle control (0.04 ± 0.01 µg/mL, *P* = 0.0029). Mice injected with unconjugated CD33 (2.268 ± 2.17 µg/mL) did not have significantly higher CD33-specific IgGs compared to PBS (*P* = 0.4062, *N* = 4–5, Fig. 2B). Since we did not detect a pharmacodynamic immune response (i.e., plasma anti-CD33 IgGs) until 3 weeks after the first dose, and survival endpoints start to be reached around this time, we first tested M2T-CD33 in a prophylactic model. We followed the same dosing schedule of 0.1 nmol M2T-CD33 or controls given every other week for three doses and then challenged mice with C1498-hCD33 cells. A significant anti-CD33 antibody response was demonstrated after the third dose (Fig. 2C). Importantly, this immune response was accompanied by an anti-AML response, as mice in the M2T-CD33 group showed significant improvement in survival after only three prophylactic doses (Fig. 2D). Cycles of biweekly dosing after injection of cells elicited even better responses with cures (defined as survival at 84 days) being observed in 90% of mice (Fig. 2E).

### Characterization of the M2T-CD33 induced immune response

**Fig. 3:**
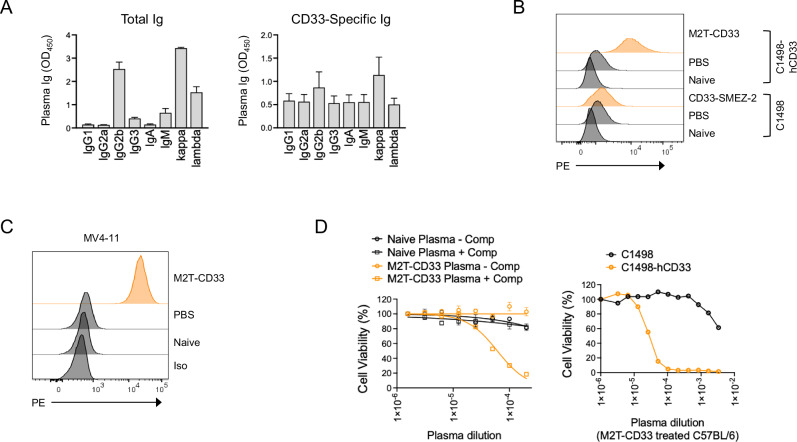
Characterization of M2T-CD33 induced antibodies showed all immunoglobulin subclasses and in vitro cytotoxicity. **A** Total and anti-CD33-specific immunoglobin isotypes present in mouse plasma after treatment with M2T-CD33 (*N* = 3). Error bars represent SEM. **B** Flow cytometry of C1498 parental and C1498-hCD33 cells incubated with Naïve, PBS treated, or M2T-CD33 treated mouse plasma. **C** Flow cytometry of MV4-11 cells incubated with Naïve, PBS treated, or M2T-CD33 treated mouse plasma. **D** Complement dependent cytotoxicity (CDC) assay with C1498-hCD33 cells incubated with baby rabbit complement and plasma from Naïve or M2T-CD33 treated mice (left). CDC assay with C1498 parental or C1498-hCD33 cells incubated with baby rabbit complement and plasma from M2T-CD33 treated mice (right).

Because MHCII plays a central role in coordinating the multiple cellular components of the immune system, we next set out to characterize the immune effects of M2T-CD33. Isotyping of total peripheral blood immunoglobulins (Igs) demonstrated that IgG2b was the dominant Ig isotype in C57BL/6 J mice, which is consistent with previous reports [50] (Fig. 3A). However, the CD33-specific antibodies induced by M2T-CD33 were more evenly distributed across the entire repertoire of Igs, including all IgG subtypes analyzed (IgG1, IgG2a, IgG2b, and IgG3), IgA, IgM, and both kappa and lambda light chains (Fig. 3A). Antibodies showed high specificity for CD33 as IgGs from treated mouse plasma bound with high affinity and specificity to C1498-hCD33 cells, but not CD33-negative parental C1498 cells, ex vivo (Fig. 3B). Importantly, IgGs generated in response to M2T-CD33 also recognized native CD33 endogenously expressed on the cell surface of human AML cells (Fig. 3C). In addition to binding, Igs were functionally active in recruiting immune-related cytotoxic effector functions. Co-incubation of C1498-hCD33 cells with blood plasma from M2T-CD33 treated mice, but not naïve mice, induced complement dependent cytotoxicity (CDC) in a CD33-dependent manner (Fig. 3D). Overall, these data demonstrate that M2T-CD33 induces a prominent CD33-specific polyclonal humoral response that utilizes the full spectrum of Ig isotypes, recognizes natively expressed CD33 on human AML cells, and has cytotoxic function against CD33-positive AML cells.

**Fig. 4:**
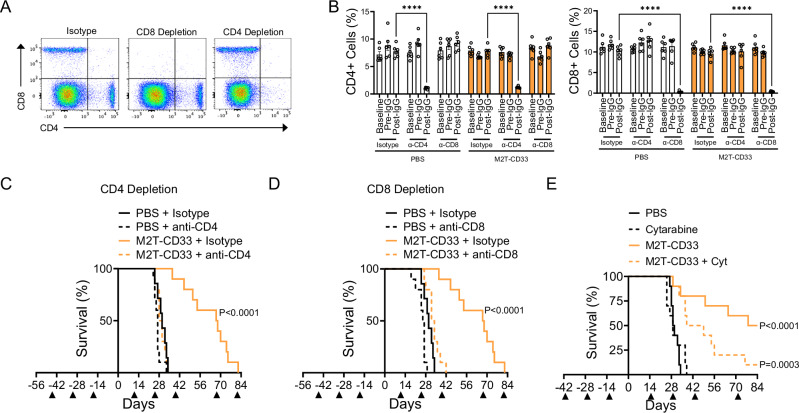
M2T-CD33 anti-AML efficacy is dependent on both CD4+ and CD8+ T cells. **A**−**D** Mice were treated with 5 µg M2T-CD33 or control as indicated by triangles and challenged with 0.5 × 10^6^ C1498-hCD33 cells (Day 0). Two days before injection of cells, depletion began with isotype control, anti-CD4, or anti-CD8 antibody. **A** Representative flow cytometry of mouse peripheral blood after treatment with isotype control, anti-CD4, or anti-CD8 antibody. CD4/CD8 percentages determined for CD45+ cells. **B** Quantification of flow cytometry on mouse peripheral blood for CD4+ and CD8+ T cell percentages from baseline, before antibody treatment (after treatment with PBS or M2T-CD33), and after two doses of antibody treatment (*N* = 6). Error bars represent SEM. Significance determined by two-way ANOVA and Tukey’s multiple comparisons test, ****P < 0.0001. **C** Kaplan-Meier survival curves for CD4+ T cell depletion (*N* = 7–10). Significance was determined by log-rank Mantel-Cox test, compared to control. **D** Kaplan-Meier survival curves for CD8+ T cell depletion (*N* = 7–10). Significance was determined by log-rank Mantel-Cox test, compared to control. **E** Mice were treated with 5 µg M2T-CD33 or control as indicated by triangles and challenged with 0.5 × 10^6^ C1498-hCD33 cells (Day 0). Cytarabine was given on Days 1, 2, 3, and 4. Kaplan-Meier survival curves are shown (*N* = 10). Significance was determined by log-rank Mantel-Cox test, compared to control.

We next evaluated the contribution of cellular immunity in the M2T-CD33 anti-leukemic response. To evaluate the role of T cells, we depleted CD4+ and CD8+ lymphocytes using mouse-specific antibodies (Fig. 4A). We treated mice first with M2T-CD33 and then depleted CD4+ or CD8+ cells prior to injection of AML cells. We used this treatment sequence in order to isolate the role of CD4+ and CD8+ cells in the anti-AML effect rather than the initial immune response against CD33, since CD4+ cells are involved in antibody isotype switching. Mice in both PBS (control) and M2T-CD33 treatment groups showed near complete depletion of CD4+ and CD8+ T cells after two doses of antibody treatment, and depletion was maintained throughout the duration of the experiment (Fig. 4B). We found that depletion of either CD4+ or CD8+ cells significantly reduced the survival improvements of M2T-CD33 (Fig. 4C, D), demonstrating a critical role of T cell immunity in the therapeutic response. Secondary observations were that CD8+ depletion by itself accelerated AML disease progression (*P* = 0.0068, *N* = 7–10), whereas there was a similar trend for CD4+ depletion that did not reach statistical significance (*P* = 0.0643, *N* = 7–10). On the other hand, CD4+ depletion completely negated any survival benefit when combined with M2T-CD33 (median survival of 27 versus 29 days, *P* = 0.057, *N* = 10), whereas the CD8+ depletion group still experienced a modest but statistically significant increase in survival upon treatment with M2T-CD33 (median survival of 26.5 versus 33 days, *P* < 0.0001, *N* = 9–10). While it is clear from this data that both CD4+ and CD8+ cells play a critical role in M2T-CD33 mediated anti-leukemic activity, it suggests that CD4+ cells have a more prominent role than CD8+ cells. We went on to evaluate the activity of M2T-CD33 in combination with SOC chemotherapy. Cytarabine (Ara-C) is a DNA damaging nucleoside analog that has been a cornerstone agent in induction regimens for the treatment of AML for decades [51], and is well-known for its lymphodepleting effects. We combined M2T-CD33 with cytarabine and found that while M2T-CD33 significantly improved survival over cytarabine treatment alone (median survival of 29 versus 43.5 days, *P* = 0.0028, *N* = 10), it also reduced the efficacy of M2T-CD33 alone (median survival 81 days versus 43.5 days, *P* = 0.0390, *N* = 10, Fig. 4E). These findings demonstrate a combination effect of M2T-CD33 and cytarabine, a standard of care agent for AML. They also point to the key role that chemotherapy-sensitive lymphocytes play in the therapeutic response to M2T-CD33, with the caveat that lymphodepleting chemotherapy drugs are likely to dampen the anti-AML efficacy of the immunotherapy.

### M2T-CD33 mounts an immune response against full-length and truncated CD33

**Fig. 5:**
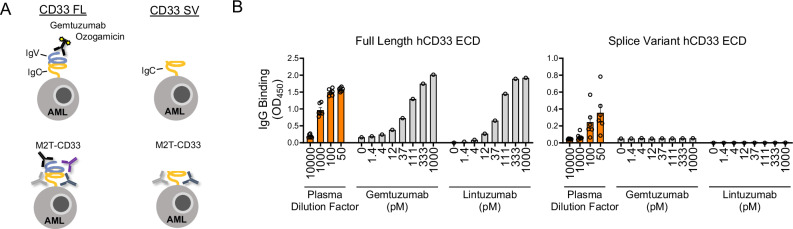
M2T-CD33 treatment induces antibodies that bind to both full length CD33 and CD33 splice variant extracellular domains (ECDs). **A** Schematic depicting full length and splice variant CD33 ECDs and binding capabilities of gemtuzumab ozogamicin and M2T-CD33 induced antibodies. **B** ELISA of plasma from M2T-CD33 treated mice (*N* = 6), gemtuzumab, and lintuzumab binding to full length CD33 ECD or CD33 splice variant ECD. Error bars represent SEM.

A limitation of existing CD33-directed therapeutics is that they bind an epitope on the CD33 IgV ECD, which is lost due to an alternative splicing event in ~50% of AML patients [29, 52]. M2T-CD33 delivers both extracellular domains of CD33, -IgV and -IgC, to MHCII for presentation to the immune system and generates a polyclonal response. We hypothesized that this would overcome the limitation of IgV-targeted drugs, as the immune system would generate antibodies against epitopes spanning the entire ECD of CD33, and not just IgV (Fig. 5A). To test this theory, we cloned and expressed both the full-length CD33 ECD and the IgC domain only to mimic the truncated protein that is expressed in AML with the splice variant. Using ELISAs, we found that blood plasma from mice treated with M2T-CD33 showed dose-dependent binding with full length CD33 ECD. This was also seen with the anti-CD33 antibodies, gemtuzumab and lintuzumab, which we produced recombinantly (Fig. S1D). Gemtuzumab is the CD33-targeting IgG4 used in gemtuzumab ozogamicin, and lintuzumab is a monoclonal that has been tested extensively in clinical trials [53]. By comparison, only IgGs from M2T-CD33 treated mice showed binding to the truncated CD33 IgC protein. Neither gemtuzumab nor lintuzumab showed any detectable binding to IgC, confirming that they recognize an epitope on the IgV domain. This further proves our hypothesis that M2T-CD33 overcomes the limitation of these two antibodies and targets both the IgV and IgC domains of CD33 (Fig. 5B).

### M2T-CD33 is well-tolerated in preclinical models

**Fig. 6:**
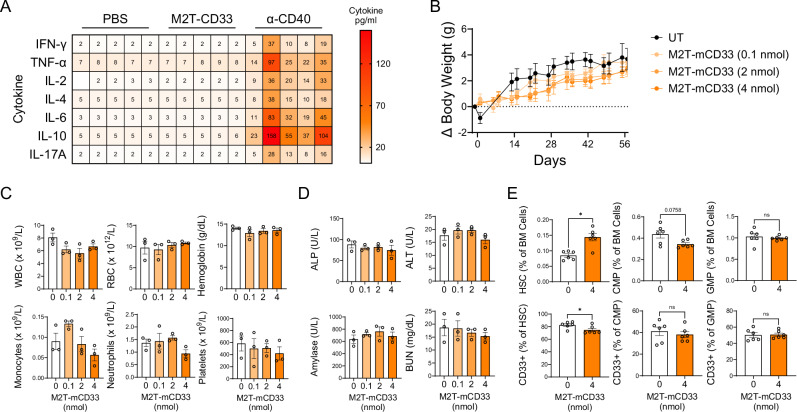
M2T-CD33 treatment is safe and tolerable in mice. **A** Mice were treated with 5 µg M2T-CD33 or PBS every other week for three doses. Plasma was taken 1 week after the third dose. Plasma from mice treated with anti-CD40 antibody was used as a positive control. Heatmap of plasma cytokine levels measured by cytometric bead array. **B**−**D** Mice were treated with the mouse version of M2T-CD33 at 0.1 nmol, 2 nmol, or 4 nmol every other week for a total of three doses and untreated mice were used as a control (*N* = 3). Error bars represent SEM. **B** Change in body weight throughout M2T-mCD33 treatment. **C** Complete blood count with differential from mice 1 week after third M2T-mCD33 dose. **D** Plasma chemistry analysis from mice 1 week after third M2T-mCD33 dose. **E** Mice were treated with 4 nmol of M2T-mCD33 or PBS every other week for a total of three doses. Quantification of flow cytometry showing frequencies of indicated populations in total live bone marrow cells 1 week after third M2T-mCD33 treatment (*N* = 6). Error bars represent SEM. Significance was determined by Mann-Whitney test, *P < 0.05, ns=not significant.

We next evaluated the safety and tolerability of M2T-CD33 in mice. First, we ensured that the genetic modifications made to M2T eliminated the immune toxicity associated with wild-type SAgs. We evaluated levels of various cytokines in plasma from M2T-CD33 treated mice and confirmed that M2T-CD33 did not induce significant changes in Th1, Th2, or Th17 cytokines, including those associated with cytokine release syndrome (CRS; Fig. 6A) [54]. This suggests that the anti-leukemic activity of M2T-CD33 is due to a targeted immune response rather than a non-specific and generalized hyper-T cell response typical of native SAgs. It further demonstrates that M2T-CD33 fails to induce a cytokine storm or the CRS that has been documented with other immunotherapy modalities [55]. We next focused on the CD33 portion of the conjugate. CD33 is a myeloid restricted surface antigen that is expressed at higher levels on AML blasts compared to normal myeloid and progenitor cells [56, 57]. However, because it is expressed on normal myeloid lineage cells, we evaluated potential hematological side effects of M2T-CD33, given prior reports of thrombocytopenia with hemorrhagic events and other cytopenias in patients treated with GO [58, 59]. For these studies we utilized an M2T-CD33 construct using the mouse CD33 ECD (AAs 18-240, M2T-mCD33) to identify off-tumor adverse effects of mounting an immune response against CD33 in rodents. We escalated the dose of M2T-mCD33 to 200 µg (4 nmol), which is 40-fold the efficacious dose we used in previous studies. We found that similar to the human targeted therapeutic molecule, M2T-mCD33 induced a robust dose-dependent mCD33-specific humoral response (Fig. S1E). Mice exhibited no overt signs of toxicity, including lethargy, changes in food or water consumption, or body condition scoring, and we observed no difference in body weight between groups over the course of the experiment (Fig. 6B). Complete blood count analysis with differential revealed no significant decreases in white or red blood cells in any of the M2T-mCD33 groups compared to the untreated control. While there was a trend towards lower levels of monocytes and neutrophils with higher doses of therapeutic, the decrease was not significant, suggesting that M2T-CD33 acts preferentially on cells with CD33 overexpression and not physiological levels (Fig. 6C, Fig. S2A). Additionally, various markers of liver, kidney, and pancreatic function all showed no changes with M2T-mCD33 treatment (Fig. 6D, Fig. S3A). Treatment showed no significant negative impact on granulocyte-macrophage progenitors (GMPs) or common myeloid progenitors (CMPs), even though both populations exhibited CD33 expression in agreement with previous reports [48]. Interestingly, there was a significant increase in hematopoietic stem cells (HSCs), but a decrease in CD33-positive HSCs, suggesting a compensatory mechanism to replenish depleted CD33+ HSCs (Fig. 6E, Fig. S3B). Overall, these data suggest a favorable preclinical safety profile for M2T-CD33.

### M2T-CD33 shows anti-AML activity in a therapeutic model and is enhanced by combination with immune checkpoint inhibition

**Fig. 7:**
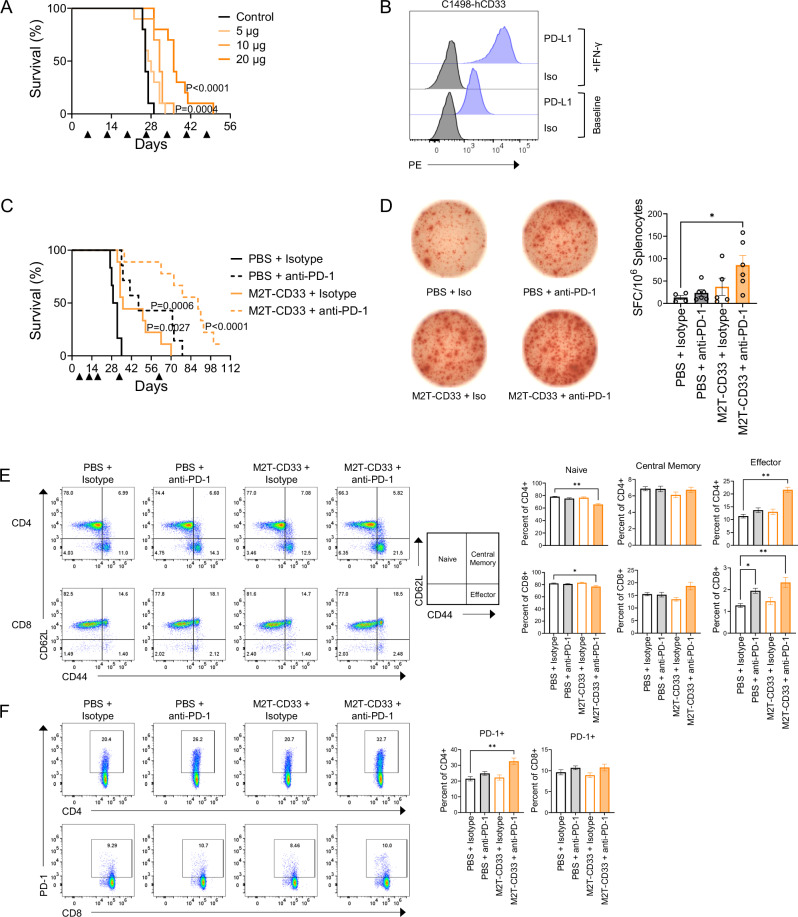
M2T-CD33 has anti-AML efficacy in an in vivo therapeutic model and in combination with immune checkpoint inhibition. **A** Mice were injected with 0.05 × 10^6^ C1498-hCD33 (Day 0) cells and the next day weekly treatment with a range of M2T-CD33 doses or PBS control began as indicated by triangles (*N* = 10). Kaplan-Meier survival curves shown. Survival significance was determined by log-rank Mantel-Cox test, compared to control. (**B**) Flow cytometry of PD-L1 expression on C1498-hCD33 cells with and without IFN-γ stimulation. **C** Mice were injected with 0.05 × 10^6^ C1498-hCD33 (Day 0) and the next day treatment with 20 µg M2T-CD33 or PBS control began as indicated by triangles. Anti-PD-1 or isotype control antibody was given twice weekly for three weeks and then once weekly after a two week break until Day 78 (*N* = 6-9). Survival significance was determined by log-rank Mantel-Cox test, compared to control. **D**−**F** Mice were treated once weekly with 20 µg M2T-CD33 or PBS control and twice weekly with anti-PD-1 or isotype control antibody for three weeks. Three weeks later, mice were challenged with 0.5 × 10^6^ C1498-hCD33 cells. One week after the injection of cells, the therapeutic regimen was repeated for 2 weeks and then splenocytes were harvested for analysis (*N* = 4–6). **D** Representative photos of IFN-γ ELISpot wells showing the hCD33-specific T cell response in splenocytes with quantification on right. Error bars represent SEM. Significance determined by Kruskal-Wallis test and Dunn’s multiple comparisons test, *P < 0.05. **E** Representative flow cytometry of splenocytes for CD44/CD62L gating within CD4+ or CD8+ T cells with quantification on right. Error bars represent SEM. Significance determined by Kruskal-Wallis test and Dunn’s multiple comparisons test, *P < 0.05, **P < 0.01. **F** Representative flow cytometry of splenocytes for PD-1 gating within CD4+ or CD8+ T cells with quantification on right. Error bars represent SEM. Significance determined by Kruskal-Wallis test and Dunn’s multiple comparisons test, **P < 0.01.

Additional in vivo efficacy studies were conducted using a treatment sequence that modeled the therapeutic rather than prophylactic setting. For these studies, mice were first injected with C1498-hCD33 AML cells and then treatments were initiated. Because of the lag in immunity onset of 21 days and the aggressiveness of the C1498 model (median survival of ~30 days), these therapeutic design experiments had a narrow window of opportunity. Nonetheless, M2T-CD33 treated mice showed significant survival improvements in a dose-dependent manner (Fig. 7A). Immune checkpoint inhibitors (ICIs), such as anti-PD-1 antibodies, have demonstrated success in a variety of tumor types, but have shown mixed results in AML [60, 61]. Consistent with previous reports [62], we found that C1498 cells express the PD-1 ligand, PD-L1, and expression increased when cells were exposed to IFN-γ (Fig. 7B). We hypothesized that combining a mouse-specific anti-PD-1 ICI with M2T-CD33 could neutralize inhibitory checkpoint blockade in T cells, widen the therapeutic window in our model, and extend survival. Anti-PD-1 therapy modestly but significantly improved animal survival as a single agent. The most dramatic improvement in survival was observed for the combination of anti-PD-1 therapy with M2T-CD33, which improved median survival to 89 days compared to 30.5 days in the control group (*P* < 0.0001, *N* = 6−9, Fig. 7C). To understand the mechanism underlying this synergy, we conducted T cell recall assays to evaluate the presence of antigen-specific T cell clones in splenocytes from mice in the different treatment cohorts. In the M2T-CD33 group, 2 out of 5 mice mounted a hCD33-specific T cell response, demonstrating the presence of antigen specific T cell clones in the spleens of treated mice. The addition of the anti-PD-1 antibody increased the number of responders to 5 out of 6 mice and showed a significant increase in the total number of IFN-γ spot-forming cells (SFC) compared to control mice (*P* = 0.0341, *N* = 4–6, Fig. 7D). We also conducted T cell immunophenotyping by multicolor flow cytometry to further understand the synergy between M2T-CD33 and anti-PD-1. In single agent M2T-CD33 treated mice, there was a trend towards increased effector CD4+ and CD8+ T cells (CD44^+^/CD62L^-^), which was further enhanced by combination with anti-PD-1 (CD4+ *P* = 0.0015; CD8+ *P* = 0.0047, *N* = 5-6). There was also a trend towards increased central memory CD8+ T cells in the combination group (Fig. 7E). We observed an increase in PD-1 expression in the anti-PD-1 treatment groups (Fig. 7F), presumably due to the increase in T cell activation and consistent with PD-1 being a dual marker of T cell activation and exhaustion [63]. By comparison, other immune checkpoint molecules, LAG-3 and TIM-3, were not induced, further emphasizing the significance of PD-1 in our model and in combination with M2T-CD33 (Fig. S4A-B). In conclusion, M2T-CD33 shows promising efficacy in an aggressive therapeutic model of AML and works synergistically with existing immunotherapies such as anti-PD-1 ICIs. This synergy further demonstrates the T cell dependent effects of M2T-CD33 and suggests potential for anti-PD-1 therapy in PD-L1-positive AML models.

## Discussion

The immune system is a complex network of both innate and adaptive cells and molecules with distinct yet complementary roles. At the center of that network are MHCI and MHCII, the major antigen presentation complexes that educate effectors and align their function against a common antigen. Our focus in this study was MHCII since its expression is restricted to professional APCs and its direct communication with CD4+ helper T cells supports the function of virtually every immune cell type. Without stimulation of the MHCII/CD4 axis, immune responses are weak or non-existent, short-lived, and ineffective. This led us to pursue therapeutic strategies that could engage MHCII and deliver tumor antigens to APCs to generate a targeted anti-tumor immune response. Previous work with the SMEZ-2 SAg, a naturally occurring protein derived from *Streptococcus pyogenes*, demonstrated MHCII binding properties along with its amenability to conjugation and genetic modification [38]. We built on these initial reports to create M2T-CD33 as a therapeutic molecule to stimulate adaptive immunity against AML. The SAg molecule was modified to ablate TCR binding while maintaining MHCII-specific binding. This strategy eliminates the APC:TCR crosslinking that is the mechanism responsible for the toxic hyperstimulation of T cells by native SAgs. The MHCII binding interface was intentionally left intact to provide a vehicle for delivering tumor associated antigens to MHCII-positive APCs for presentation to the immune system. Our experiments systematically show that conjugating CD33 ECD to a modified SMEZ-2 SAg elicits an antigen-specific anti-AML response in a syngeneic mouse model. We showed that M2T-CD33 has significant anti-AML efficacy in both prophylactic and therapeutic models. We further demonstrated that the anti-AML mechanism includes a polyclonal humoral response and both CD4+ and CD8+ T cells. Studies combining M2T-CD33 with the AML SOC chemotherapy agent Ara-C/cytarabine and an anti-PD-1 antibody showed promising combination activity, positioning M2T-CD33 for clinical trials as both a single agent and in combination with existing SOC and other immunotherapies. It is important to note that M2T-CD33 efficacy is dependent on a functional immune system, given its reliance on MHCII engagement and subsequent presentation of CD33 to immune effector cells. AML patients often present with dysfunctional hematopoiesis due to uncontrolled proliferation of leukemic blasts in the bone marrow compartment and immunosuppression caused by conventional chemotherapy. Careful consideration to this potential obstacle will be required in the design of M2T-CD33 clinical trials. Additional experiments with other in vivo AML models are warranted, particularly models that reflect important elements of human AML, including heterogeneity, immune complexity, and CD33 expression dynamics.

CD33 is an appealing target for AML immunotherapy, as its expression is limited to the myeloid compartment, it is cell surface localized and accessible to the humoral immune system, and it has a high density of expression in 90% of AML cases. One CD33-targeted FDA approved drug, GO, has demonstrated efficacy in AML. One major disadvantage of GO is the design of its monoclonal antibody, gemtuzumab, which binds to an epitope on the IgV domain of CD33. This design flaw presumably neutralizes its efficacy in the 50% of AML patients that express a CD33 splice variant lacking the IgV domain. Though it has been theorized, to our knowledge, our study is the first to provide experimental evidence that gemtuzumab is unable to bind to the recombinant IgC domain of CD33. We also demonstrated the same result for lintuzumab, a CD33 monoclonal antibody that was tested previously in AML clinical trials [53, 64], but was abandoned by the sponsor. This is, as far as we are aware, also the first study to demonstrate that lintuzumab is incapable of binding to truncated CD33, information which may be important for ongoing clinical studies of lintuzumab radioligand conjugates [65, 66]. M2T-CD33 induces a polyclonal immune response with a diverse repertoire of Igs that bind to multiple epitopes spanning the IgV and IgC domains of the CD33 ECD. Based on this, we propose that M2T-CD33 would exhibit efficacy across all CD33-positive AML cases, including adult and pediatric AML, regardless of CD33 population polymorphism. While we demonstrated the binding superiority of M2T-CD33 induced IgGs over gemtuzumab and lintuzumab, future head-to-head studies are required to fully demonstrate superior anti-leukemic activity of M2T-CD33 in AML models.

M2T-CD33 was well tolerated with a favorable safety profile. CRS is a safety concern associated with newer immunotherapy modalities, and we did not observe any evidence of CRS in M2T-CD33 treated mice. We hypothesize this is due to the gradual and coordinated adaptive immune response that M2T induces through MHCII, which is fundamentally different from the immune effects of CAR-T and bispecific T cell engagers that induce an acute burst of T cell activity immediately upon administration. We also evaluated potential toxicities associated with CD33 using a construct that specifically targets the mouse CD33 protein. Clinical studies with GO have provided insight into the adverse events associated with targeting CD33 in humans. Veno-occlusive disease (VOD) is a serious and potentially fatal side effect associated with GO. However, VOD may be attributed to the calicheamicin rather than an effect of targeting CD33, as VOD is also observed in patients treated with inotuzumab ozogamicin, which utilizes calicheamicin in conjugation with and an anti-CD22 antibody [5, 30, 67]. Importantly, we observed no signs of hepatotoxicity with M2T-CD33 even with doses 40X higher than the efficacious dose. Hematological toxicity was another common side effect of GO, presumably driven by on-target off-tumor activity of targeting CD33 on normal myeloid cells, which has been observed with other CD33-targeted therapeutics [68, 69]. Aside from a trend toward reduced neutrophil and monocyte counts, we did not observe major hematological toxicity in M2T-CD33 treated mice. Additionally, we showed that CD33-expressing CMP and GMP myeloid progenitor populations were spared by the immune response induced by M2T-CD33. We hypothesize this is due to the significantly higher expression density of CD33 on AML cells compared to their normal myeloid cell counterparts [57]. Overall, these safety studies demonstrate early safety signals for M2T-CD33. While promising, these initial findings in murine models should be confirmed in humanized mouse models and higher animal species, and these studies are currently being planned by our group.

Beyond M2T-CD33, the M2T platform is a versatile, off-the-shelf therapeutic platform that can be adapted for multiple tumor antigens and cancer types. Its advantages include the potential to target tumor antigens directly to MHCII on APCs. Other approaches that attempt to force presentation of tumor antigens through MHC molecules on APCs have focused on peptide and mRNA-based approaches that use computational methods to deliver antigens that are predicted to bind to and be presented by MHCI and MHCII [70, 71]. A drawback of these approaches is the poor accuracy of MHCII theoretical models, which have lagged behind MHCI [72, 73]. The fidelity of MHCII predictions is limited by several factors including the complex structure of the MHCII binding groove, incomplete understanding of how tumor antigens are processed through the MHCII pathway, and the highly polymorphic nature of the multiple MHCII genes (HLA-DR, -DP, and -DQ) [74]. Another limitation of peptide and mRNA-based approaches is that they rely on stochastic forces for molecules to reach MHCII-expressing APCs. There is thus an opportunity and need for a novel approach that directly targets the MHCII complex and forces antigen presentation to the immune system, uses a tried-and-true manufacturing process and distribution chain, and is independent of patient tumor and HLA genetic diversity. The M2T platform may offer all of these advantages, and our study demonstrates the potential of M2T-CD33 for the treatment of AML.

## Supplementary information

M2T-CD33 Supplementary Figure Legends

Supplementary Figure 1

Supplementary Figure 2

Supplementary Figure 3

Supplementary Figure 4

## Data Availability

Data are available from the corresponding author upon reasonable request.
