# Supplementary material for: A superantigen-based MHC class II-targeted cancer immunotherapy for the treatment of acute myeloid leukemia
Source: Blood Cancer J. 2025 Nov 17;15(1):198. doi: 10.1038/s41408-025-01391-w (PMC12623995; doi:10.1038/s41408-025-01391-w)
Supplement: Supplementary file 1 — M2T-CD33 Supplementary Figure Legends [file 41408_2025_1391_MOESM1_ESM.docx]

**Supplemental Figure Legends**

**Figure S1.**

(A) Aggregation assay of SMEZ-2, CD33, and M2T-CD33 before and after amino acid modifications. Error bars represent SEM. Significance was determined by one-way ANOVA with Sidak’s multiple comparisons test, **P<0.01, ***P<0.001, ns=not significant.

(B) Flow cytometry plots of human PBMCs (healthy donor #2) gating on CD3 or CD19 and then comparing binding of CD33 vs. M2T-CD33.

(C) Flow cytometry of mouse AML cell lines showing endogenous CD33 expression.

(D) Flow cytometry of CD33-negative Jurkat and C1498-hCD33 cells incubated with recombinant Gemtuzumab (Gem), left. Flow cytometry of C1498 parental and C1498-hCD33 cells incubated with recombinant Lintuzumab (Lin), right.

(E) ELISA of anti-CD33 IgG one week after third M2T-mCD33 dose. Error bars represent SEM. Significance determined by Kruskal-Wallis test and Dunn’s multiple comparisons test, *P<0.05.

**Figure S2.**

(A) Mice were treated with the mouse version of M2T-CD33 at 0.1 nmol, 2 nmol, or 4 nmol every other week for a total of three doses and untreated mice were used as a control (N=3). Complete blood count with differential from mice one week after third M2T-mCD33 dose.

**Figure S3.**

(A) Mice were treated with the mouse version of M2T-CD33 at 0.1 nmol, 2 nmol, or 4 nmol every other week for a total of three doses and untreated mice were used as a control (N=3). Plasma chemistry analysis from mice one week after third M2T-mCD33 dose.

(B) Flow cytometry gating strategy of mouse bone marrow to analyze HSC, CMP, and GMP populations.

**Figure S4.**

(A)-(B) Mice were treated once weekly with 20 µg M2T-CD33 or PBS control and twice weekly with anti-PD-1 or isotype control antibody for three weeks. Three weeks later, mice were challenged with 0.5 x 10^6^ C1498-hCD33 cells. One week after the injection of cells, the therapeutic regimen was repeated for two weeks and then splenocytes were harvested for analysis (N=4-6).

(A) Representative flow cytometry of splenocytes for LAG-3 gating within CD4+ or CD8+ T cells with quantification on right. Error bars represent SEM.

(F) Representative flow cytometry of splenocytes for TIM-3 gating within CD4+ or CD8+ T cells with quantification on right. Error bars represent SEM. Significance determined by Kruskal-Wallis test and Dunn’s multiple comparisons test, *P<0.05.
