# Supplementary material for: A superantigen-based MHC class II-targeted cancer immunotherapy for the treatment of acute myeloid leukemia
Source: Blood Cancer J. 2025 Nov 17;15(1):198. doi: 10.1038/s41408-025-01391-w (PMC12623995; doi:10.1038/s41408-025-01391-w)
Supplement: Supplementary file 2 — Supplementary Figure 1 [file 41408_2025_1391_MOESM2_ESM.pptx]

## Slide 1
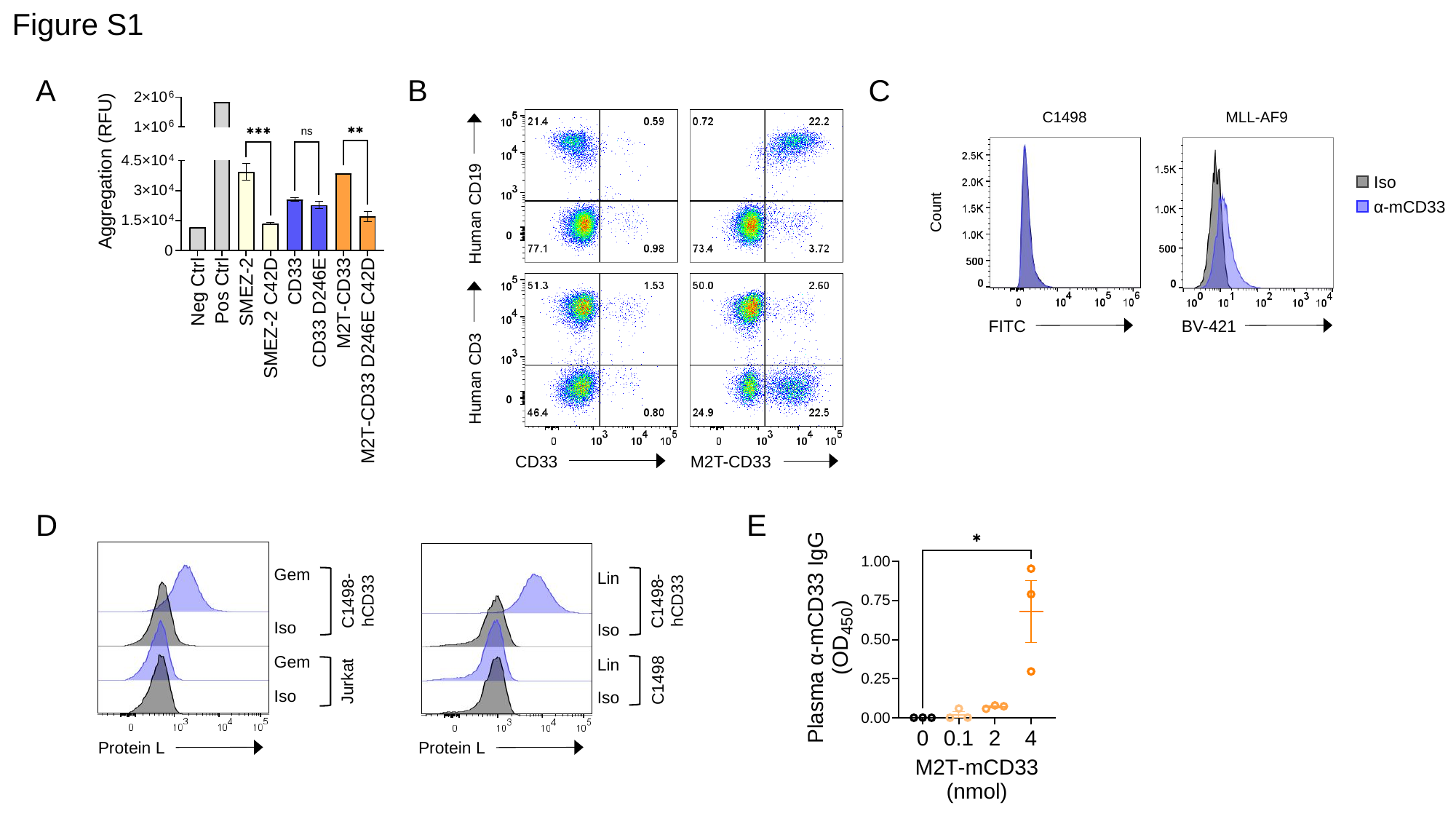

Figure S1
A
B
C
Human CD19
Human CD3
CD33
M2T-CD33
C1498
MLL-AF9
Iso
α-mCD33
Count
FITC
BV-421
D
E
Gem
C1498-hCD33
Iso
Gem
Jurkat
Iso
Protein L
Lin
C1498-hCD33
Iso
Lin
C1498
Iso
Protein L
