# Supplementary figures and images for: A superantigen-based MHC class II-targeted cancer immunotherapy for the treatment of acute myeloid leukemia
Source: Blood Cancer J. 2025 Nov 17;15(1):198. doi: 10.1038/s41408-025-01391-w (PMC12623995; doi:10.1038/s41408-025-01391-w)

## Slide 1
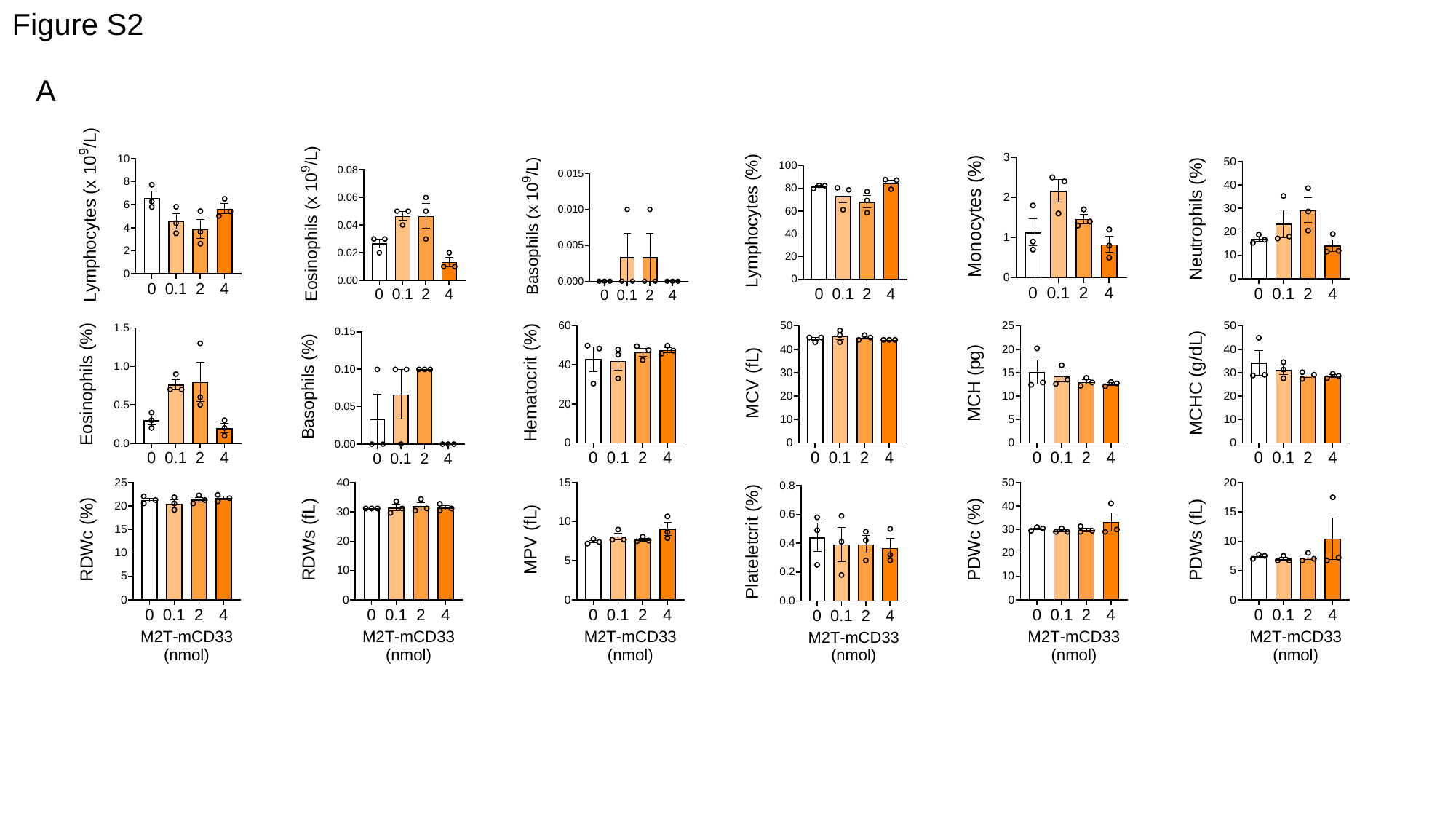

Figure S2
A

Supplement: Supplementary file 3 — Supplementary Figure 2 [file 41408_2025_1391_MOESM3_ESM.pptx]

## Slide 1
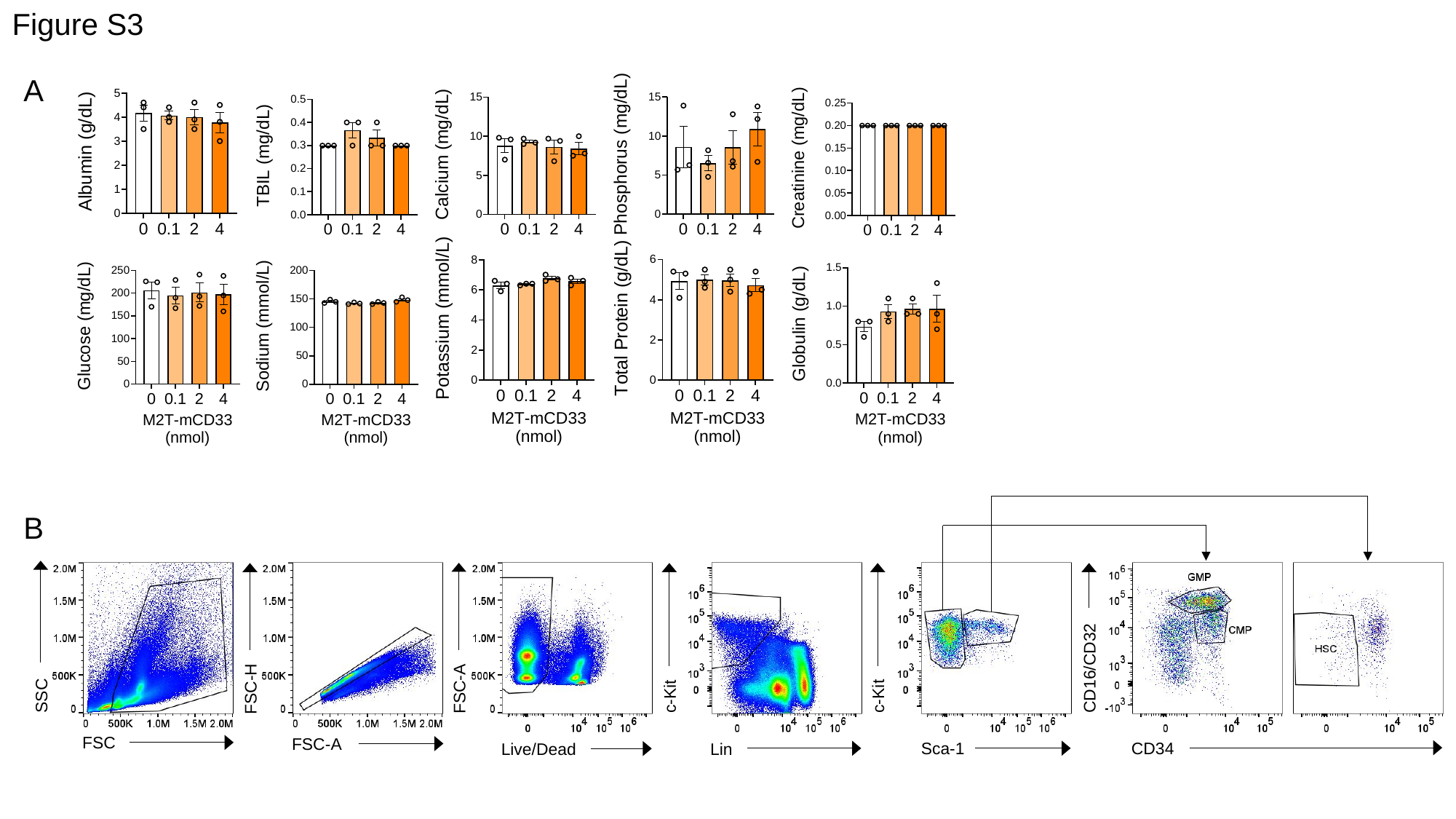

Figure S3
A
CD16/CD32
FSC-A
FSC-H
SSC
c-Kit
c-Kit
FSC
FSC-A
CD34
Sca-1
Lin
Live/Dead
B

Supplement: Supplementary file 4 — Supplementary Figure 3 [file 41408_2025_1391_MOESM4_ESM.pptx]
