# Supplementary material for: A superantigen-based MHC class II-targeted cancer immunotherapy for the treatment of acute myeloid leukemia
Source: Blood Cancer J. 2025 Nov 17;15(1):198. doi: 10.1038/s41408-025-01391-w (PMC12623995; doi:10.1038/s41408-025-01391-w)
Supplement: Supplementary file 5 — Supplementary Figure 4 [file 41408_2025_1391_MOESM5_ESM.pptx]

## Slide 1
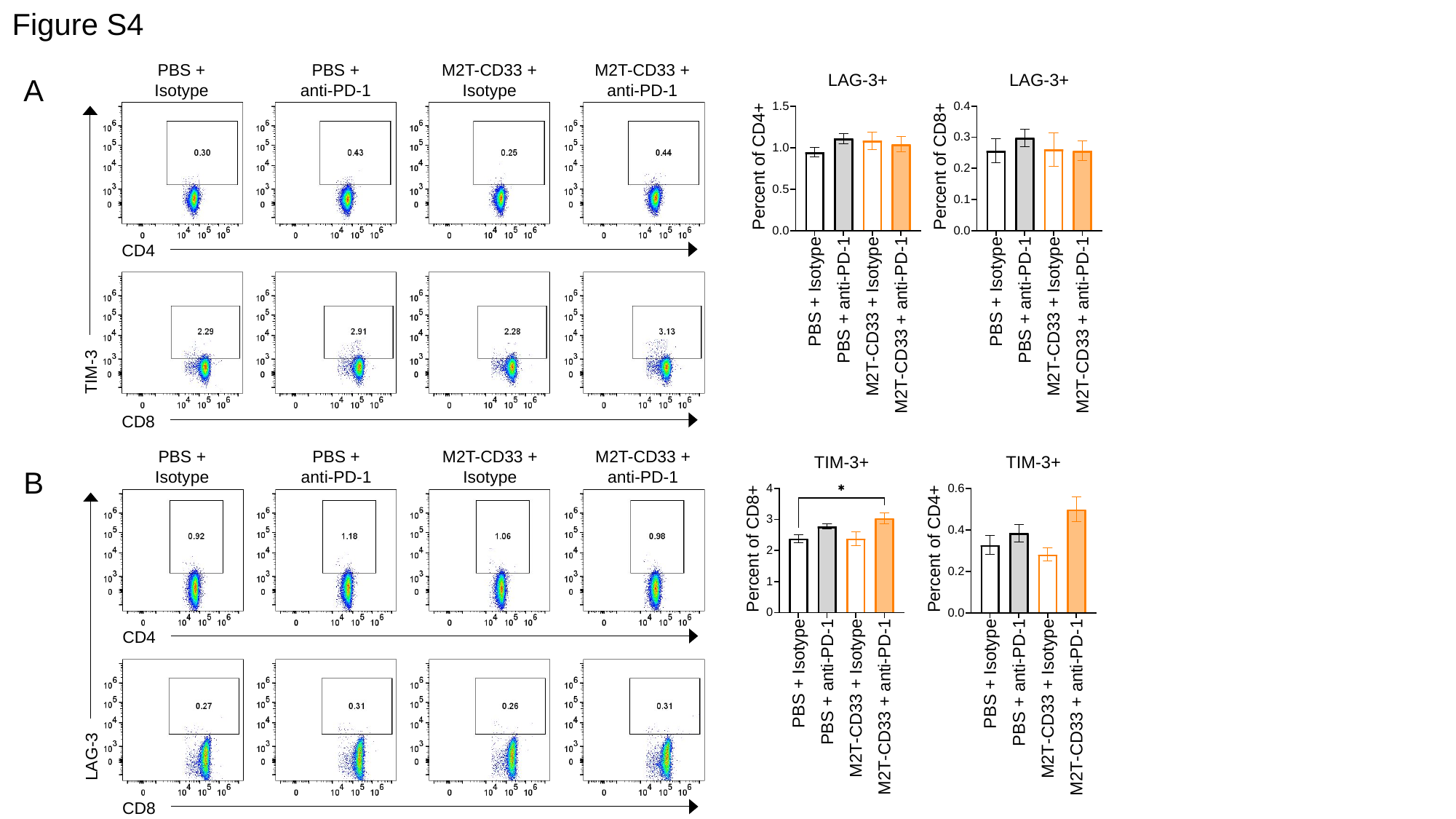

Figure S4
PBS + Isotype
PBS + anti-PD-1
M2T-CD33 + Isotype
M2T-CD33 + anti-PD-1
CD4
TIM-3
CD8
A
PBS + Isotype
PBS + anti-PD-1
M2T-CD33 + Isotype
M2T-CD33 + anti-PD-1
CD4
LAG-3
CD8
B
